# Transactions South Carolina Medical Association

**Published:** 1874-09

**Authors:** 


TRANSACTIONS OF THE SOUTH CAROLINA MEDICAL ASSOCIATION. Annual session held in Columbia, April 14th and 15th, 1874.

Appendix: Annual address by the President; report on osteogenesis and reproduction of bone, by W. H. Bailey, M.D.; report of the committee on the effects upon the fœtus of the protracted use of chloroform during parturition, by Dr. F. M. Robertson, chairman; excission of superior maxillary bone for fibro-sarcomatous tumor, cure, by J. S. Buist, M.D., (illustrated;) miscellaneous cases in private practice, by Francis L. Parker, M.D., (illustrated;) removal of knife blade from the brain, by E. B. Turnipseed, M. D.; Cases of Guinea worm, by J. S. Buist, M.D.; hernia within the funicular portion of the processus vaginalis—strangulation, excission of sac with testicle, radical cure, by R. W. Gibbes, M.D.; twin pregnancy, abortion in second month, and natural birth at full time, by W. T. C. Bates, M.D.; case of bright's disease, recovery, by E. B. Turnipseed, M.D.; traumatic epilepsy ten years, operation thirteen years, after injury, by John T. Darby, M.D., (illustrated;) operation for congenital harelip, by R. W. Gibbes, M.D.

